# Contemporary economic burden in a real‐world heart failure population with Commercial and Medicare supplemental plans

**DOI:** 10.1002/clc.23585

**Published:** 2021-03-11

**Authors:** Carolyn S. P. Lam, Robert Wood, Muthiah Vaduganathan, Hector Bueno, Alex Chin, Gabriela Luporini Saraiva, Elisabeth Sörstadius, Theo Tritton, Joseph Thomas, Lei Qin

**Affiliations:** ^1^ National Heart Centre Singapore & Duke‐National University of Singapore Medical School Singapore; ^2^ University Medical Centre Groningen The Netherlands; ^3^ Adelphi Real World Bollington UK; ^4^ AstraZeneca Gaithersburg (Consultant) Maryland USA; ^5^ Division of Cardiovascular Medicine Brigham and Women's Hospital & Harvard Medical School Boston Massachusetts USA; ^6^ Centro Nacional de Investigaciones Cardiovasculares (CNIC) Madrid Spain; ^7^ Department of Cardiology Hospital Universitario 12 de Octubre and Instituto de Investigación Sanitaria Hospital 12 de Octubre (imas12) Madrid Spain; ^8^ CIBER de Enfermedades CardioVasculares (CIBERCV) Madrid Spain; ^9^ Facultad de Medicina Universidad Complutense de Madrid Madrid Spain; ^10^ AstraZeneca Gothenburg Sweden

**Keywords:** cost, ejection fraction, healthcare resource utilization, heart failure, real world

## Abstract

**Background:**

Limited real‐world data exist on healthcare resource utilization (HCRU) and associated costs of patients with heart failure (HF) with reduced ejection fraction (HFrEF) and preserved EF (HFpEF), including urgent HF visits, which are assumed to be less burdensome than HF hospitalizations (hHFs)

**Hypothesis:**

This study aimed to quantify the economic burden of HFrEF and HFpEF, via a retrospective, longitudinal cohort study, using IBM® linked claims/electronic health records (Commercial and Medicare Supplemental data only).

**Methods:**

Adult patients, indexed on HF diagnosis (ICD‐10‐CM: I50.x) from July 2012 through June 2018, with 6‐month minimum baseline period and varying follow‐up, were classified as HFrEF (I50.2x) or HFpEF (I50.3x) according to last‐observed EF‐specific diagnosis. HCRU/costs were assessed during follow‐up.

**Results:**

About 109 721 HF patients (22% HFrEF, 31% HFpEF, 47% unclassified EF; median 18 months' follow‐up) were identified. There were 3.2 all‐cause outpatient visits per patient‐month (HFrEF, 3.3; HFpEF, 3.6); 69% of patients required inpatient stays (HFrEF, 80%; HFpEF, 78%). Overall, 11% of patients experienced hHFs (HFrEF, 23%; HFpEF, 16%), 9% experienced urgent HF visits (HFrEF, 15%; HFpEF, 12%); 26% were hospitalized less than 30 days after first urgent HF visit versus 11% after first hHF. Mean monthly total direct healthcare cost per patient was $9290 (HFrEF, $11 053; HFpEF, $7482).

**Conclusions:**

HF‐related HCRU is substantial among contemporary real‐world HF patients in US Commercial or Medicare supplemental health plans. Patients managed in urgent HF settings were over twice as likely to be hospitalized within 30 days versus those initially hospitalized, suggesting urgent HF visits are important clinical events and quality improvement targets.

## 1 INTRODUCTION

Heart failure (HF) is an important cause of mortality and morbidity,^1^ yet has broader health implications, including substantial economic burden on healthcare systems. In the context of shifting HF epidemiology with rising projected disease burden, safely curbing HF‐related costs has emerged as a common goal for patients and healthcare systems. Patients may seek acute HF care in non‐hospitalization settings, including emergency departments, HF clinics, observation units, urgent‐care centers, and ambulatory infusion sites.^2^, ^3^ Increasing HF prevalence^4^ is expected to drive HF‐related direct costs to $53 billion by 2030.^5^ Despite recognition of the economic burden of HF, limited data exist estimating the impact on healthcare resource utilization (HCRU) and direct medical costs of HF management across care settings. Even less information exists on cost and HCRU variation according to left ventricular ejection fraction (LVEF), specifically patients with HF with preserved ejection fraction (HFpEF) or reduced ejection fraction (HFrEF), despite increasing awareness of the burden of HFpEF.^6^ The primary study aim was to estimate HCRU and associated direct medical costs, including HF hospitalizations (hHFs) and urgent HF visits, in a contemporary HF‐patient cohort. Secondary aims were estimation of HCRU/costs by LVEF‐specific diagnosis, and comparison of HCRU/cost outcomes by age and prior/recent inpatient stay.

## 2 METHODS

This was a retrospective, longitudinal cohort study of a prevalent HF population using linked US claims and electronic healthcare records (EHRs) data between 2012 and 2018.

**FIGURE 1:**
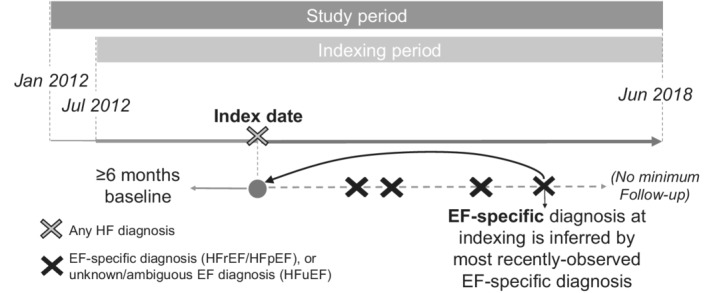
Study design. EF, ejection fraction; HF, heart failure; HFpEF, heart failure with preserved ejection fraction; HFrEF, heart failure with reduced ejection fraction; HFuEF, heart failure with unclassified ejection fraction

Adult patients were indexed on date of first/earliest claim with an HF diagnosis code^7^ (ICD‐9/10‐CM 428.x/I50.x) from July 2012 through June 2018. Continuous medical and pharmacy eligibility for ≥6 months before indexing (baseline period) was required to capture baseline demographics and clinical characteristics. Variable follow‐up extended from indexing until the earliest of loss of medical/pharmacy eligibility or end of study period, that is, ranging from 0 to 71 months (Figure 1).

Patients with distinct forms of cardiomyopathy (ICD‐9/10‐CM 425.x/I42.x‐43.x) during baseline were excluded. Patients were classified as HFrEF (ICD‐9/10‐CM 428.2x/I50.2x) or HFpEF (ICD‐9/10‐CM 428.3x/I50.3x) at indexing according to the last‐observed LVEF‐specific diagnosis during follow‐up, including indexing, in the expectation that patients undergo further testing/examination over time, leading to greater ability to make LVEF‐specific diagnoses. Patients without an LVEF‐specific diagnosis (i.e., LVEF not measured/recorded) or ambiguously labeled (i.e., combined HFrEF/HFpEF diagnosis) were classified as HF with unclassified ejection fraction (HFuEF).

This retrospective analysis involved no decisions on patient interventions and patient‐level data were anonymized. Institutional review board/ethics approval and patient informed consent were not required.

Linked claims (MarketScan Commercial and Medicare Supplemental) and EHR (Explorys) data collated by IBM® Watson Health were used (see Appendix S1). The 5‐year Limited Claims‐EHR Dataset (LCED) was used—a static dataset covering ≈4.4 million patients from January 2012 through June 2018 (claims) and September 2018 (EHRs).

All‐cause HCRU and expenditures were reported by service type (inpatient, outpatient, and pharmaceutical). HCRU/costs associated with HF‐related medications, hHFs, and urgent HF visits were derived separately. The hHFs were defined as hospitalizations with ≥1 overnight stay and primary diagnosis of HF. Urgent HF visits were defined as emergency department visits with HF as the primary diagnosis, but not constituting an hHF. Resource users were patients using ≥1 unit of a given healthcare service.

Two sensitivity analyses regarding LVEF status classification were conducted: (a) using diagnosis at index only, and (b) using last‐observed LVEF‐specific diagnosis, omitting patients with conflicting LVEF‐specific diagnoses during follow‐up. These are described further in the Appendix S1.

This was a descriptive study. Outcomes were stratified by LVEF subgroup (HFpEF, HFrEF, and HFuEF), age at indexing (18–49, 50–64, and ≥65 years), and baseline inpatient stay (Y/N). Statistical significance was assessed via univariate regression models accounting for variable follow‐up: negative binomial/Poisson regression models for count outcomes, generalized linear models (with log link and gamma distribution) for cost outcomes, and complementary log–log models for dichotomous outcomes. The *p*‐values <.05 were considered statistically significant.

Statistical analyses were conducted in Stata 16 (StataCorp LP, College Station, Texas).

## 3 RESULTS

The study cohort consisted of 109 721 eligible patients with HF (HFrEF, 22%; HFpEF, 31%; HFuEF, 47%) (Figure S1). Among the HFuEF subgroup (n = 51 984), 92% did not receive an LVEF‐specific diagnosis during follow‐up and 8% received a combined HFrEF/HFpEF diagnosis. This article focuses on the HFrEF and HFpEF subgroups because of the ambiguity of HFuEF diagnoses. The mean age at index was 73 years and 50% were men (Table 1). Median follow‐up was 18 months (HFrEF, 21 months; HFpEF, 20 months). The most frequently reported comorbidities were hypertension (82%), type 2 diabetes (41%), and depression/anxiety/cognitive disorders (34%).

**TABLE 1:** Baseline patient demographics and clinical characteristics

Characteristic	All (n = 109 721)	Left ventricular ejection fraction		
		rEF(n = 23 956)	pEF(n = 33 781)	uEF(n = 51 984)
Mean age (SD), years	72.8 (14)	72.6 (13)	74.9 (12)	71.5 (15)
Sex				
Male	54 312 (50%)	14 554 (61%)	14 320 (42%)	25 438 (49%)
Female	55 409 (50%)	9402 (39%)	19 461 (58%)	26 546 (51%)
Healthcare plan type				
Comprehensive	40 325 (37%)	9207 (38%)	13 331 (39%)	17 787 (34%)
Health maintenance organization	29 211 (27%)	5905 (25%)	9202 (27%)	14 104 (27%)
Preferred provider organization	34 719 (32%)	7663 (32%)	10 158 (30%)	16 898 (33%)
Other plan type	10 964 (10%)	2439 (10%)	2725 (8%)	5800 (11%)
Median length of follow‐up (IQR), months	17.9 (6.7–35.8)	21.0 (8.8–39.7)	20.4 (8.5–38.6)	15.3 (5.2–31.8)
Comorbidity				
Hypertension	89 540 (82%)	18 863 (79%)	29 188 (86%)	41 489 (80%)
T2DM	44 947 (41%)	10 137 (42%)	14 730 (44%)	20 080 (39%)
Depression, anxiety, and cognitive disorders	37 671 (34%)	6656 (28%)	12 030 (36%)	18 985 (37%)
Atrial fibrillation	31 121 (28%)	7386 (31%)	10 534 (31%)	13 201 (25%)
Peripheral artery/vascular disease	30 044 (27%)	6354 (27%)	10 073 (30%)	13 617 (26%)
CKD	23 764 (22%)	5198 (22%)	8364 (25%)	10 202 (20%)
Anemia (iron deficiency)	20 770 (19%)	3983 (17%)	7145 (21%)	9642 (19%)
Obesity	20 086 (18%)	3523 (15%)	7319 (22%)	9244 (18%)
Cancer	19 827 (18%)	4124 (17%)	6110 (18%)	9593 (19%)
Sleep apnea	19 369 (18%)	3616 (15%)	6938 (21%)	8815 (17%)
Cerebrovascular disease/stroke	17 244 (16%)	3284 (14%)	5756 (17%)	8204 (16%)
Acute coronary syndrome/myocardial infarction	14 906 (14%)	4187 (17%)	4161 (12%)	6558 (13%)
Hyperkalemia/hypokalemia	14 421 (13%)	2598 (11%)	4966 (15%)	6857 (13%)
Pulmonary hypertension	2376 (2%)	417 (2%)	970 (3%)	989 (2%)
Mean (SD) baseline Charlson Comorbidity Index	2.0 (2.2)	1.9 (2.1)	2.1 (2.1)	1.9 (2.3)
BMI	n = 46 407	n = 9613	n = 15 216	n = 21 578
Mean (SD)	30.4 (7.9)	29.8 (7.3)	31.2 (8.2)	30.1 (7.8)
Baseline systolic BP, mm Hg	n = 47 330	n = 9800	n = 15 441	n = 22 089
Mean (SD)	134 (21)	133 (21)	136 (21)	133 (21)
Baseline diastolic BP, mm Hg	n = 47 303	n = 9793	n = 15 433	n = 22 077
Mean (SD)	73 (12)	74 (12)	73 (12)	73 (12)
Baseline eGFR, mL/min/1.73 m^2^	n = 28 507	n = 5855	n = 9650	n = 13 002
Mean (SD)	55.5 (26.3)	55.1 (25.2)	54.5 (24.4)	56.5 (27.9)
Baseline HbA_1c_, %	n = 19 400	n = 4091	n = 6651	n = 8658
Mean (SD)	6.8 (1.5)	6.9 (1.6)	6.8 (1.5)	6.7 (1.5)
Baseline BNP, pg/mL	n = 10 012	n = 2067	n = 3776	n = 4169
Mean (SD)	325 (823)	444 (1029)	303 (890)	285 (611)
Baseline *N*‐terminal proBNP, pg/mL	n = 3183	n = 636	n = 1204	n = 1343
Mean (SD)	2160 (4737)	2895 (5273)	2040 (4713)	1918 (4451)

*Notes*: The *P* < .0001 for all comparisons across LVEF subgroups.

Abbreviations: BMI, body mass index; BNP, B‐type natriuretic peptide; BP, blood pressure; CKD, chronic kidney disease; eGFR, estimated glomerular filtration rate; HbA_1c_, glycated hemoglobin; IQR, interquartile range; pEF, preserved ejection fraction; proBNP, pro‐brain natriuretic peptide; rEF, reduced ejection fraction; SD, standard deviation; T2DM, type 2 diabetes mellitus; uEF, unclassified ejection fraction.

Baseline characteristics were numerically similar between HFrEF and HFpEF, except for age (HFrEF, 73 years; HFpEF, 75 years), sex (HFrEF, 61% men; HFpEF, 42% men), and comorbidities (generally more prevalent in HFpEF).

Beta‐blockers (58%; HFrEF 74%, HFpEF 61%) and angiotensin‐converting enzyme inhibitor/angiotensin receptor blockers (45%; HFrEF 56%, HFpEF 47%) were the most frequently dispensed guideline‐directed medical therapy (GDMT); sodium‐glucose cotransporter‐2 inhibitors (SGLT2is) and angiotensin receptor‐neprilysin inhibitors (ARNIs) were dispensed to 1.0% (93% with prior diabetes diagnosis) and 0.5% of the study cohort, respectively; 39% (HFrEF 56%, HFpEF 41%) received ≥2 GDMT classes (Figure S2). GDMT use was higher in patients with HFrEF versus HFpEF for all therapy classes. Other frequently dispensed classes included diuretics and statins (both 52%). Notable significant differences were observed between HFrEF and HFpEF in dispensing of calcium channel blockers (23% vs. 35%) and mineralocorticoid receptor antagonists (24% vs. 14%). Diuretic use was similar between HFrEF (63%) and HFpEF (62%).

The rate of outpatient visits was 3.2 per patient‐month in the study cohort (HFrEF 3.3, HFpEF 3.6; Table 2). The most‐visited outpatient service providers were acute‐care hospitals (18% of single‐day visits) and family practitioners (16%). Two‐thirds (69%) of the study cohort required an inpatient stay; the rate of inpatient stays was 0.07 per patient‐month and was comparable between HFrEF and HFpEF (both 0.08). HF was the most frequently recorded primary diagnosis across all inpatient stays (13% of stays); other diagnoses were “other sepsis” (6%), “acute myocardial infarction” (4%), and “atrial fibrillation and flutter” (4%). The mean (SD) length of stay (LoS) was 6.0 (6.3) days and mean (SD) inpatient LoS across entire follow‐up was 14.3 (21.9) days; LoS did not differ substantially between HFrEF and HFpEF.

**TABLE 2:** Healthcare resource utilization and direct costs during the follow‐up period

Resource*	All (n = 109 721)	Left ventricular ejection fraction		
		rEF(n = 23 956)	pEF(n = 33 781)	uEF(n = 51 984)
All‐cause resource use				
Outpatient				
No. of outpatient visits, mean (SD)	71.8 (91.6)	83.0 (98.3)	88.2 (105.4)	56.0 (74.5)
Incidence rate (95% CI)	3.176(3.165–3.187)	3.312(3.308–3.317)	3.597(3.593–3.601)	2.766(2.763–2.769)
Most commonly used outpatient resources, visits (% responses)				
Total frequency of events	7 020 026	2 672 350	4 020 627	327 049
Acute‐care hospital	1 246 236 (18%)	485 627 (18%)	697 564 (17%)	63 045 (19%)
Family practice	1 089 894 (16%)	426 725 (18%)	614 788 (17%)	48 381 (15%)
Internal medicine (NEC)	520 997 (7%)	194 912 (7%)	301 967 (8%)	24 118 (7%)
Supply center	428 275 (6%)	151 159 (6%)	258 718 (6%)	18 398 (6%)
Cardiovascular disease/cardiology	372 439 (5%)	174 327 (7%)	182 421 (5%)	17 744 (5%)
Radiology	312 863 (4%)	116 000 (4%)	180 368 (4%)	14 442 (4%)
Treatment center	284 280 (4%)	114 748 (4%)	156 899 (4%)	12 633 (4%)
Home help agency	197 596 (3%)	70 170 (3%)	119 446 (3%)	7980 (3%)
Unknown	188 580 (3%)	66 991 (3%)	114 133 (3%)	7456 (2%)
Laboratory	171 284 (2%)	68 310 (3%)	95 074 (2%)	7900 (2%)
Inpatient	*n = 75 705*	*n = 19 276*	*n = 26 207*	*n = 30 222*
No. of hospital admissions, mean (SD)	1.6 (2.0)	2.0 (2.3)	2.0 (2.3)	1.1 (1.5)
Incidence rate (95% CI)	0.070(0.069–0.070)	0.081(0.081–0.082)	0.083(0.082–0.083)	0.053(0.052–0.053)
Mean (SD) LoS/hospitalization	6 (6.3)	5.9 (5.8)	5.8 (5.1)	6.3 (7.4)
Mean (SD) cumulative LoS	14.3 (21.9)	15.7 (23.0)	16.0 (22.9)	12.1 (20.0)
Reasons for admission	*n = 124 654*	*n = 49 267*	*n = 69 804*	*n = 5583*
HF	15 673 (13%)	7583 (15%)	7419 (11%)	671 (12%)
Other sepsis	8042 (6%)	2857 (6%)	4773 (7%)	412 (7%)
Acute myocardial infarction	5381 (4%)	3170 (6%)	1851 (3%)	360 (6%)
Atrial fibrillation and flutter	5233 (4%)	2285 (5%)	2727 (4%)	221 (4%)
Other chronic obstructive pulmonary disease	4568 (4%)	1366 (3%)	3050 (4%)	152 (3%)
Respiratory failure (NEC)	4225 (3%)	1367 (3%)	2654 (4%)	204 (4%)
Acute kidney failure	3986 (3%)	1468 (3%)	2357 (3%)	161 (3%)
Pneumonia, unspecified organism	3723 (3%)	1332 (3%)	2257 (3%)	134 (2%)
Hypertensive heart and chronic kidney disease	2811 (2%)	1197 (2%)	1492 (2%)	122 (2%)
Chronic ischemic heart disease	2701 (2%)	1431 (3%)	1129 (2%)	141 (3%)
Cerebral infarction	2297 (2%)	935 (2%)	1235 (2%)	127 (2%)
Other	66 014 (53%)	24 276 (49%)	38 860 (56%)	2878 (52%)
HF‐related resource use				
Urgent visits (all patients)				
Mean no. (SD)	0.1 (0.6)	0.3 (0.8)	0.2 (0.7)	0 (0.3)
Incidence rate (95% CI)	0.006(0.006–0.006)	0.010(0.010–0.010)	0.008(0.008–0.008)	0.002(0.002–0.003)
Urgent visits (resource users)	*n = 10 000*	*n = 3583*	*n = 4214*	*n = 2203*
Mean no. (SD)	1.5 (1.2)	1.7 (1.3)	1.6 (1.2)	1.2 (0.5)
Incidence rate (95% CI)	0.056(0.055–0.057)	0.057(0.055–0.058)	0.053(0.052–0.054)	0.062(0.060–0.065)
hHFs (all patients)*				
Mean no. (SD)	0.1 (0.5)	0.3 (0.7)	0.2 (0.6)	0 (0.2)
Incidence rate (95% CI)	0.006(0.006–0.006)	0.012(0.012–0.013)	0.009(0.009–0.009)	0.001(0.001–0.001)
hHFs (resource users)	*n = 12 252*	*n = 5544*	*n = 5549*	*n = 1159*
Mean no. (SD)	1.3 (0.7)	1.3 (0.7)	1.3 (0.8)	1.1 (0.3)
Incidence rate (95% CI)	0.048(0.047–0.049)	0.048(0.047–0.049)	0.046(0.045–0.047)	0.059(0.056–0.062)
Mean (SD) LoS (all hHF events)	5.2 (6.0)	5.3 (5.5)	5.1 (6.3)	5.1 (7.2)
Mean (SD) cumulative LoS	6.8 (8.5)	7.1 (8.4)	6.8 (8.7)	5.6 (8.1)
Direct medical costs, mean (SD)*				
Medication, all	10 723 (31256)	11 363 (30082)	12 537 (34960)	9249 (29091)
HF medication				
All	1577 (5555)	2009 (4112)	1886 (1886)	1176 (2957)
Resource users	1941 (6106)	2327 (4341)	2214 (9301)	1541 (3301)
Outpatient visits, all	39 730 (105902)	48 909 (114597)	45 427 (114075)	31 799 (95185)
Urgent visits				
All	104 (881)	201 (1219)	141 (1109)	35 (382)
Resource users	1141 (2709)	1346 (2897)	1130 (2958)	828 (1667)
Inpatient stays, all	40 317	54 386	44 292	31 250
hHFs				
All	2578	6536	3171	368
Resource users	23 084	28 243	19 304	16 505

*Notes*: The *P < .0001 for all comparisons across LVEF subgroups.

Abbreviations: CI, confidence interval; HF, heart failure; hHF, heart failure hospitalization; LoS, length of stay; NEC, not elsewhere classified; pEF, preserved ejection fraction; rEF, reduced ejection fraction; SD, standard deviation; uEF, unclassified ejection fraction.

**FIGURE 2:**
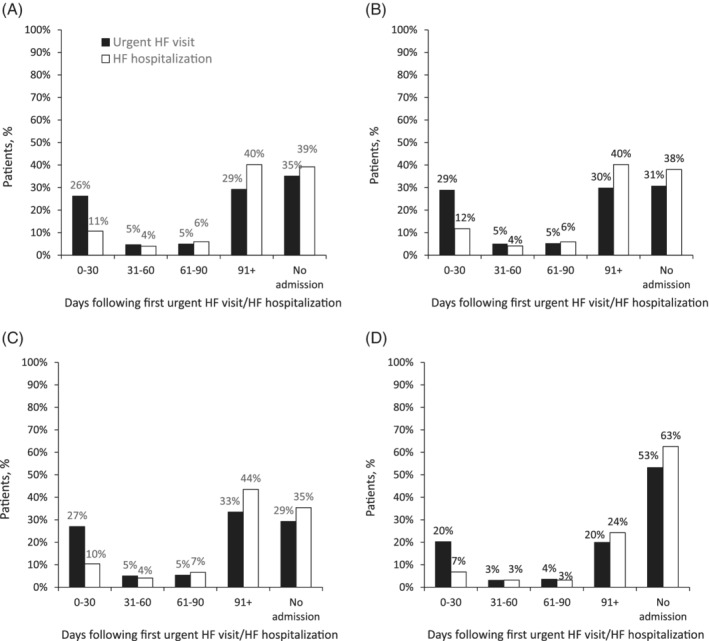
Subsequent all‐cause hospitalization after hHF or urgent HF visit: (A) all patients; (B) HFrEF; (C) HFpEF; (D) HFuEF. HF, heart failure; HFpEF, heart failure with preserved ejection fraction; HFrEF, heart failure with reduced ejection fraction; HFuEF, heart failure with unclassified ejection fraction; hHF, heart failure hospitalization. *P* < .05 for all HFrEF/HFpEF comparisons with the following exceptions: readmission after hHF within 31–60 days and 61–90 days; admitted after an urgent HF visit within 31–60 days, 61–90 days, and not admitted

In total, 9% of patients had an urgent HF visit (HFrEF, 15%; HFpEF, 12%; Table 2). The rate of urgent HF visits among resource‐users was 0.06 per patient‐month (HFrEF 0.06, HFpEF 0.05). One‐quarter (26%) of patients were hospitalized (all‐cause) within 30 days of initial urgent HF visit (HFrEF, 29%; HFpEF, 27%), with 65% of patients hospitalized any time following the urgent HF visit (HFrEF, 69%; HFpEF, 71%; Figure 2). Furthermore, 11% of all patients had an hHF during follow‐up (HFrEF, 23%; HFpEF, 16%; Figure 2). The rate of hHFs among resource users was 0.05 per patient‐month, with a mean LoS of 5.2 days (numerically similar for HFrEF and HFpEF). Among all resource users, the rate of hospitalizations requiring ≥1 overnight stay with secondary diagnosis of HF (i.e., not an hHF) was 0.07 per patient‐month, with a mean LoS of 7.0 days. In total, 11% of all patients were readmitted (all‐cause) within 30 days of their first hHF (HFrEF, 12%; HFpEF, 10%); 61% were subsequently readmitted at some time during follow‐up (HFrEF, 62%; HFpEF, 65%).

**FIGURE 3:**
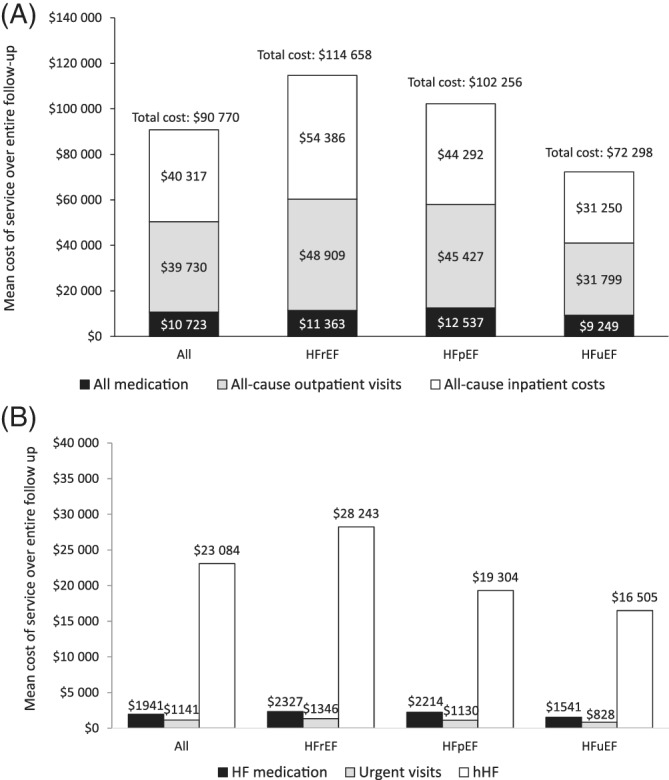
Costs associated with healthcare resource use in patients with HF: (A) All cause and (B) HF (resource users). *P* < .0001 for all HFrEF/HFpEF comparisons with the following exceptions: HF medication (*P* = NS), urgent HF visits (*P* = NS). HF, heart failure; HFpEF, heart failure with preserved ejection fraction; HFrEF, heart failure with reduced ejection fraction; HFuEF, heart failure with unclassified ejection fraction; hHF, heart failure hospitalization

All‐cause costs associated with HF management by LVEF are shown in Table 2 and Figure 3(A). The mean total healthcare cost per patient (monthly cost per patient) was $90 770 ($9290); $114 658 ($11 053) for HFrEF, and $102 256 ($7482) for HFpEF. The total medication cost per patient was $10 723 ($457); ≈12% of total healthcare costs (5% of total monthly costs). Although total medication costs were higher for HFpEF versus HFrEF ($12 537 [$495] and $11 363 [$429]), HF‐related medication costs were higher for HFrEF compared with HFpEF ($2009 [$4112] and $1886 [$1886]).

The total cost of outpatient visits per patient (all cause) was $39 730 ($2395); 44% of total healthcare costs (26% of total monthly costs). Outpatient costs were $48 909 ($2603) for HFrEF and $45 427 ($2318) for HFpEF. The cost of inpatient stays per patient was $40 317 ($6438); 44% of total healthcare costs (69% of total monthly costs). Inpatient stays cost $54 386 (monthly $8021) for HFrEF and $44 292 ($4668) for HFpEF.

HF costs, reported per resource‐using patient, included HF medication costs of $1941 ($82) (Figure 3(B)); $2327 ($90) for HFrEF and $2214 ($87) for HFpEF. The cost of urgent HF visits was $1141 ($122); $1346 ($88) for HFrEF and $1130 ($108) for HFpEF. hHF costs per patient were $23 084 ($2754); $28 243 ($3372) for HFrEF and $19 304 ($1916) for HFpEF. The cost of inpatient stays with only a secondary diagnosis of HF was $8904 ($1435).

HCRU by age is summarized in Table S1. LoS of hHFs was significantly longer in younger patients; these patients also had significantly shorter times to readmission than older patients. Total healthcare costs (all cause) and HF costs were also higher in younger patients.

Medication use was similar among patients who had and had not required hospitalization at baseline (data not shown). Outpatient visits and inpatient stays were more common in previously hospitalized patients; LoS was also significantly longer (Table S1). The number of urgent HF visits and hHFs was numerically similar across subgroups. Previously hospitalized patients had higher total healthcare costs (all cause) compared with those not previously hospitalized, predominantly driven by inpatient stays and outpatient visits (Table S1).

HCRU and costs by underlying T2DM and CKD at indexing are summarized in Table S2. Mean total healthcare costs and many components of HCRU were significantly higher for patients with versus without T2DM or CKD.

Sensitivity analyses using index diagnosis only or excluding patients with conflicting LVEF‐specific diagnoses during follow‐up, produced similar results for cost data as primary study analyses (Figure S3). Results were directionally consistent (i.e., higher for HFrEF vs. HFpEF), and numerically similar. The proportions of costs attributed to each setting were also consistent with primary analyses.

## 4 DISCUSSION

This longitudinal cohort study of linked claims/EHRs data highlights substantial economic burden related to contemporary HF care. The average per‐patient monthly cost for healthcare was estimated at $9290, driven by high rates of inpatient and outpatient visits. Estimated costs (and most HCRU measures) were generally higher for HFrEF compared with HFpEF. Higher costs were observed among those recently hospitalized. Urgent HF visits were frequent for both HFrEF and HFpEF. Patients managed via urgent‐care settings were over twice as likely to be hospitalized for any reason within 30 days versus those managed via hHFs. Young patients with HF spent the most time in hospital and experienced shorter readmission times.

The economic cost of HF management is considerable. Hospitalizations contribute substantially to direct medical costs of HF, but other significant direct costs should be considered, including medications, procedures, nursing‐home costs, and physician appointments.^2^ Hospitalization also negatively impacts patients and families.^8^, ^9^ Although the economic cost of HF has been widely studied, few studies examined burden of HFpEF and HFrEF. HCRU and costs have been reported to be significantly greater in patients with chronic HFrEF after a worsening HF event versus patients who remain stable.^10^ This study, undertaken to quantify the real‐world economic burden of these subgroups in the US, adds to those findings and provides a more comprehensive insight into clinical profiles, HCRU, and direct medical costs of patients with HFrEF and HFpEF.

Observed HCRU was high: during follow‐up patients experienced a rate of 3.2 all‐cause outpatient visits per month: patients with HFpEF had a higher incidence rate of all‐cause outpatient visits versus HFrEF (3.6 vs. 3.3 visits per month). One in 10 patients had ≥1 urgent HF visit during follow‐up. Our study included a high proportion of patients with HFuEF, many of whom had not yet received an LVEF‐specific diagnosis, suggesting these patients may be recently diagnosed and awaiting further testing. Such patients may have had a milder or even transient disease state compared with the HFpEF and HFrEF subgroups, thereby diluting the rate of worsening HF events during follow‐up. These patients likely also contributed to underestimation of other resources and corresponding costs in the study cohort. Alternatively, these patients may simply reflect less specific diagnostic classification by the treating physician.

Two‐thirds of the study cohort were hospitalized (all‐cause) and 10% experienced ≥1 hHF. Patients with HF are frequently multimorbid and HF was not always the primary diagnosis; other diagnoses included sepsis, acute myocardial infarction, and atrial fibrillation/flutter. Total healthcare costs were high, particularly for HFrEF, driven almost equally by inpatient and outpatient costs, a finding misaligned with traditional focus on reducing financial costs for inpatient settings. Nonetheless, we found that patients with a recent hospitalization had higher HCRU than those without. Overall, these data provide support for measures to reduce costs in both care settings.

Urgent HF visits are important clinical events and our real‐world data highlight the related, substantial HCRU. A key finding was that patients managed in urgent‐care settings were more than twice as likely to be hospitalized (all‐cause) within 30 days versus those managed via an hHF (26% vs. 11%, respectively). This likely reflects that patients presenting at urgent HF visits represent a high‐risk cohort with substantial longitudinal care needs. The 30‐day readmission rate after an hHF in our study is lower than the 25% of readmissions reported for Medicare beneficiaries,^11^, ^12^ although differences in study designs, patient populations, and study definitions may account for this disparity.

The importance of urgent HF visits is increasingly being recognized. Inclusion of urgent HF visits in a sensitivity analysis of the PARAGON‐HF trial resulted in statistically significant differences in the primary outcome for a study that otherwise failed to show differences between arms.^13^ In that study, sacubitril–valsartan did not significantly lower rates of total hHFs and death from cardiovascular disease versus valsartan, although the inclusion of confirmed urgent HF visits in a composite endpoint resulted in a risk ratio of 0.861 (95% confidence interval 0.747–0.993). Notably, the DAPA‐HF study included urgent HF visits in the primary endpoint and demonstrated a reduced risk of worsening HF or death from cardiovascular causes in patients who received dapagliflozin versus placebo plus standard therapy.^14^ Urgent HF visits also represent important targets for quality improvement, which may require focused attention and resource allocation similar to investments in post‐discharge transitional care.

This real‐world US study also highlights the substantial economic burden across a broad age range, including younger patients (<65 years). Use of the LCED, primarily covering a commercial health plan including younger patients, allowed detailed description of HCRU/costs in this cohort. Total costs were highest among patients aged less than 50 years, primarily driven by longer inpatient stays, despite lower medication costs; findings supported by another US‐based study.^15^ Younger patients also experienced shorter times to readmission. Total, all‐cause monthly costs per patient were almost twice as high for patients aged less than 65 years compared with patients aged 65+ ($14 386 vs. $7335), primarily driven by inpatient costs ($10 700 vs. $4804). Monthly costs per patient for outpatient visits, medications, and all HF‐related events were higher in patients aged less than 65 years, apart from HF‐related medication costs. Others have shown that young patients with HFpEF have poor quality of life compared with older patients and are more likely to die of cardiovascular‐related causes,^16^, ^17^ emphasizing the importance of improving outcomes in these patients. Total healthcare costs were significantly higher for patients diagnosed with T2DM or CKD versus those with no T2DM or CKD, in line with findings from prior work.^18^

Utilization of GDMT was low overall, which is particularly concerning in patients with HFrEF given the strong evidentiary base supporting their clinical benefits in this patient population. Dedicated HF registries encompassing broad real‐world HF populations in the United States and worldwide (CHAMP‐HF, CHECK‐HF, REPORT‐HF, ASIAN‐HF, and BIOSTAT) have shown suboptimal use of established and newer therapies targeting HFrEF.^19^, ^20^, ^21^, ^22^, ^23^ In the present study, the proportion of patients on triple therapy (three evidence‐based HF therapies) remained less than 20% and use of ARNI and SGLT2i remained less than 2%. Various patient‐level (affordability, willingness to take multidrug regimens), clinician‐level (comfort with newer agents, knowledge gaps, treatment inertia), and health system‐level (local treatment availability, access to healthcare) issues may contribute to observed gaps in evidence‐based therapies.^19^, ^21^, ^22^, ^24^ Multilevel quality improvement initiatives are needed to promote equitable and widespread care practices to optimize GDMT.

Some study limitations should be considered. Diagnoses were identified using ICD‐9/10‐CM codes, which are subject to miscoding. Low diuretic use may indicate incomplete reporting of prescriptions, and in some cases, accuracy of HF diagnoses. Recently, LVEF thresholds for HFrEF and HFpEF have evolved,^25^ potentially causing confusion in patient diagnosis/classification.^26^ Claims‐based models aiming to better identify LVEF‐specific subgroups are being developed, which may improve characterization of HCRU in these populations.^26^ Observed statistically significant differences may be driven, in part, by large sample sizes; comparisons should emphasize absolute differences. Only US Commercial and Medicare supplemental data were evaluated, which may limit the generalizability of these findings to other healthcare systems or other covered patient populations in the United States. Finally, the large proportion of patients classified as HFuEF may have impacted study findings. This subgroup likely comprises a combination of patients with a definitive clinically valid mid‐range or borderline LVEF diagnosis, patients with misdiagnosed LVEF status, and patients without a recorded LVEF. This subgroup therefore represents a heterogenous group without an interpretable shared characteristic.

Accurate coding and LVEF‐specific diagnosis of patients may represent an opportunity for improvement in care quality. All outcomes during follow‐up were attributed to HFrEF, HFpEF, or HFuEF based upon last‐observed LVEF‐specific diagnosis; this may have resulted in misclassification of patients with multiple or borderline LVEF diagnoses and/or overestimation of HCRU/costs for these patient subgroups. Nonetheless, sensitivity analyses (based on index LVEF only, and excluding patients with conflicting LVEF‐specific diagnoses) yielded similar findings to the main analysis.

Study strengths included linkage of claims and EHRs data, which facilitated comprehensive capture of patients' healthcare interactions. A minimum follow‐up for inclusion was not specified, mitigating risk of introducing immortal person‐time bias. Code lists were developed with clinical input to ensure they accurately represented disease types and health‐related events. The 6‐month baseline period was ultimately used as a best effort to balance sample size and certainty that patient characteristics captured were accurate. Finally, the long follow‐up allowed assessment of HCRU/costs over a substantial time.

This study, one of the first to assess real‐world HCRU specific to HFrEF and HFpEF in the United States, demonstrates the substantial HCRU of patients with HFrEF and HFpEF, and quantifies HCRU related to urgent HF visits, showing that these are important clinical events representing a target for quality improvement. Future efforts are needed to understand if coordinated multidisciplinary HF clinics or other initiatives may help diffuse and/or reduce healthcare system costs. Overall, our results identify key drivers of costs among patients with HF and highlight the need for their effective management in real‐world settings.

## CONFLICTS OF INTEREST

Carolyn Lam is supported by a Clinician Scientist Award from the National Medical Research Council of Singapore; has received research support from Boston Scientific, Bayer, Roche Diagnostics, AstraZeneca, Medtronic, and Vifor Pharma; has served as consultant or on the Advisory Board/Steering Committee/Executive Committee for Boston Scientific, Bayer, Roche Diagnostics, AstraZeneca, Medtronic, Vifor Pharma, Novartis, Amgen, Merck, Janssen Research & Development LLC, Menarini, Boehringer Ingelheim, Novo Nordisk, Abbott Diagnostics, Corvia, Stealth BioTherapeutics, JanaCare, Biofourmis, Darma, Applied Therapeutics, MyoKardia, WebMD Global LLC, Radcliffe Group Ltd and Corpus, and is a co‐founder and non‐executive director of eKO.ai.

Robert Wood, Theo Tritton, and Joseph Thomas are employed by Adelphi Real World.

Muthiah Vaduganathan is supported by the KL2/Catalyst Medical Research Investigator Training award from Harvard Catalyst (NIH/NCATS Award UL 1TR002541), serves on advisory boards for Amgen, AstraZeneca, Baxter Healthcare, Bayer AG, Boehringer Ingelheim, Cytokinetics, and Relypsa, and participates on clinical endpoint committees for studies sponsored by Galmed, Novartis, and the National Institutes of Health.

Hector Bueno receives research funding from the Instituto de Salud Carlos III, Spain (PIE16/00021 & PI17/01799), Sociedad Española de Cardiología, AstraZeneca, Bayer, BMS and Novartis; has received consulting fees from AstraZeneca, Bayer, BMS‐Pfizer, Novartis; and speaking fees or support for attending scientific meetings from Amgen, AstraZeneca, Bayer, BMS‐Pfizer, Novartis, and MEDSCAPE‐the heart.org.

Alex Chin, Gabriela Luporini Saraiva, Elisabeth Sörstadius, and Lei Qin are employed by and are shareholders of AstraZeneca.

## Supporting information


**Appendix S1**: Supporting Information
