# Supplementary material for: Contemporary economic burden in a real‐world heart failure population with Commercial and Medicare supplemental plans
Source: Clin Cardiol. 2021 Mar 11;44(5):646–55. doi: 10.1002/clc.23585 (PMC8119853; doi:10.1002/clc.23585)
Supplement: Supplementary file 1 — Appendix S1: Supporting Information [file CLC-44-646-s001.docx]

Supplementary Appendix

Lam C et al. Contemporary economic burden in a real-world heart failure population with Commercial and Medicare Supplemental plans

# Expanded Methods

## Data sources

MarketScan^®^ claims datasets contain healthcare data for a convenience sample of ≥43.6 million insured individuals.^1^ The MarketScan^®^ Commercial Database comprises medical and drug data from employers and health plans and contains data for several million individuals annually, encompassing employees, their spouses, and dependents who are covered by employer-sponsored private health insurance in the US. Healthcare for these individuals is provided under a variety of fee-for-service, fully capitated, and partially capitated health plans.^1–3^ Medical claims are linked to outpatient prescription drug claims and person-level enrollment information. As these data are primarily sourced from employers, claims for mail-order prescriptions and specialty pharmacies are included to fully capture prescription fills from all locations.

The MarketScan^®^ Medicare Supplemental Database profiles healthcare experiences of retirees with Medicare supplemental insurance paid by employers and includes the Medicare-covered portion of payments, employer-paid portion, and out-of-pocket patient expenses. This database provides detailed cost, use, and outcomes data for inpatient and outpatient healthcare services. For most of the population, medical claims are linked to outpatient prescription drug claims and person-level enrollment data using unique patient or enrollee identifiers. Beneficiaries in the Medicare Supplemental Database have drug coverage; therefore, drug data are available and provide additional valuable information. Explorys^®^ is an electronic healthcare record system that acts as a fundamental record of patient interactions with the healthcare system.^2^ Explorys^®^ Universe encompasses longitudinal data originating from >55 million unique patients, covering multiple distinct healthcare systems and providers, and documenting information including healthcare encounters, procedures, vital signs, test results, and patient-reported outcomes.

MarketScan^®^ and Explorys^®^ data are linked at the patient level using a deterministic algorithm, with a reported false-positive rate of 0.015%^3^; the linked dataset was de-identified as required under Health Insurance Portability and Accountability Act provisions.

## Analysis

Healthcare costs were based on paid amounts of adjudicated claims, including insurer and health plan payments, and patient cost-sharing (copayment and coinsurance). All healthcare interventions resulting in a claim were included in the analysis. Dispensing fees for outpatient drug claims were also included.

Costs for services provided under capitated arrangements were estimated using payment proxies computed based on paid claims at procedure level (i.e., imputation of median values). All US dollar estimates were inflated to 2018 costs using Medical Care Component of the Consumer Price Index.^4^

## Sensitivity analyses

Feasibility queries highlighted that patients typically received a ‘generic’ heart failure (HF) diagnosis before a left ventricular ejection fraction (LVEF)-specific diagnosis. Using the last-observed diagnosis would reduce the number of patients with unclassified/ambiguous LVEF (i.e. HFuEF), thus increasing the HF with reduced LVEF (HFrEF)/preserved LVEF (HFpEF) subpopulations and representativeness. This method would also allow the patient to have undergone more testing/examinations and increase the likelihood of the patient receiving an LVEF-specific diagnosis from a specialist, thus increasing accuracy of the underlying diagnosis.

Feasibility queries also highlighted that some patients received both HFrEF and HFpEF diagnoses during follow-up. HFrEF is not an evolution of HFpEF, thus a significant amount of switching was not expected. Echocardiogram readings have limitations, with considerable variation regarding LVEF, thus using the last-observed diagnosis would increase accuracy of underlying diagnosis. Furthermore, the expected proportion (estimated at 5% from feasibility queries) of patients with alternative HFrEF/HFpEF diagnosis at indexing vs last observed was deemed sufficiently low so as to not affect the study findings.

Sensitivity analyses were performed to explore these alternative methods of classifying LVEF status. In sensitivity analysis 1, the patient’s diagnosis at index, rather than the last-observed diagnosis, was used. In sensitivity analysis 2, patients receiving both diagnoses (HFrEF and HFpEF) from index to the end of follow-up were excluded from the analysis.

# Results

## Sensitivity analysis

Based on patient LVEF status at diagnosis only, 12%, 20%, and 68% of patients had HFrEF, HFpEF, and HFuEF, respectively. In sensitivity analysis 1, which used the patient’s LVEF category at diagnosis, 6% (n=1,443) of existing HFrEF patients and 4% (n=1,267) of existing HFpEF patients would be diagnosed with HFrEF instead of HFpEF and vice versa using this alternative study design, resulting in a small proportion of patients with potential misdiagnosis.

For sensitivity analysis 2, 61,925 of 109,721 patients (56%) had ≥1 LVEF-specific diagnosis code recorded between index and end of follow-up (including combined HFrEF/HFpEF diagnoses). Of these, 17,812 (29%) had >1 LVEF-specific diagnosis code recorded, specifically at least 2 of HFrEF, or HFpEF, or combined HFrEF/HFpEF. A total of 9,279 of these patients (52%; 8% of full study cohort) had both an HFrEF and a HFpEF diagnosis code recorded between index and end of follow-up.

# References

1. IBM Watson Health. IBM MarketScan Research Databases for Health Services Researchers – WHITE PAPER. Available at: <https://www.ibm.com/downloads/cas/6KNYVVQ2>. Accessed December 12, 2020.
2. IBM Watson Health. IBM Explorys EPM Application Suite - SOLUTION BRIEF. Available at: <https://www.ibm.com/downloads/cas/ZZQYQQRL>. Accessed December 12, 2020.
3. IBM Watson Health. IBM Watson Health. Deterministic patient matching. Available at: <https://www.ibm.com/downloads/cas/AKZO2ANR>. Accessed December 12, 2020.
4. U.S. Bureau of Labor Statistics. Consumer Price Index for All Urban Consumers (CPI-U): U. S. city average, by expenditure category. Available at: https://www.bls.gov/news.release/cpi.t01.htm. Accessed December 12, 2020.

**SUPPLEMENTARY TABLE S1** Healthcare resource utilization during follow-up and associated costs in patients with HF stratified by age and prior hospitalization

|  | Age at index (years) | | | Hospitalized at baseline | |
| --- | --- | --- | --- | --- | --- |
| Parameter | 18–49 (n=6,189) | 50–64 (n=24,225) | 65+ (n=79,307) | No (n=63,347) | Yes (n=46,374) |
| **All-cause resource use** |  |  |  |  |  |
| Outpatient visits (all patients) |  |  |  |  |  |
| Mean no. (SD) | 55.4 (92.4) | 67.5 (94.5) | 74.4 (90.4) | 68.2 (86.0) | 76.7 (98.5) |
| Incidence rate (95% CI) | 2.636 (2.627–2.644) | 3.011 (3.006–3.015) | 3.265 (3.262–3.267) | 2.774 (2.771–2.776) | 3.855 (3.851–3.859) |
| Inpatient stays (all patients) |  |  |  |  |  |
| Mean no. (SD) | 1.5 (2.7) | 1.6 (2.4) | 1.6 (1.8) | 1.4 (1.8) | 1.8 (2.3) |
| Incidence rate (95% CI) | 0.70 (0.069–0.072) | 0.70 (0.069–0.071) | 0.069 (0.069–0.070) | 0.058 (0.058–0.058) | 0.089 (0.089–0.090) |
| **HF-related resource use** |  |  |  |  |  |
| Urgent visits (all patients) |  |  |  |  |  |
| Mean no. (SD) | 0.1 (0.4) | 0.1 (0.5) | 0.2 (0.6) | 0.1 (0.6) | 0.1 (0.6) |
| Incidence rate  (95% CI) | 0.0035 (0.0032–0.0039) | 0.0041 (0.0039–0.0042) | 0.007 (0.0069–0.0071) | 0.0054 (0.0053–0.0056) | 0.0074 (0.0072–0.0076) |
| Urgent visits (resource users) | *n=332* | *n=1,445* | *n=8,223* | *n=5,446* | *n=4,554* |
| Mean no. (SD) | 1.4 (0.8) | 1.5 (1.3) | 1.5 (1.2) | 1.6 (1.1) | 1.5 (1.2) |
| Incidence rate  (95% CI) | 0.0589 (0.0537–0.0645) | 0.0548 (0.0526–0.0572) | 0.0558 (0.0548–0.0568) | 0.0526 (0.0515–0.0538) | 0.0601 (0.0587–0.0616) |
| hHFs (all patients) |  |  |  |  |  |
| Mean no. (SD) | 0.1 (0.4) | 0.1 (0.5) | 0.2 (0.5) | 0.1 (0.5) | 0.1 (0.5) |
| Incidence rate  (95% CI) | 0.0042 (0.0039–0.0046) | 0.0052 (0.0050–0.0054) | 0.0069 (0.0068–0.0070) | 0.0061 (0.0059–0.0062) | 0.0069 (0.0067–0.0071) |
| hHFs (resource users) | *n=408* | *n=2,132* | *n=9,712* | *n=7,368* | *n=4,884* |
| Mean no. (SD) | 1.3 (0.8) | 1.3 (0.9) | 1.3 (0.7) | 1.3 (0.7) | 1.3 (0.8) |
| Incidence rate  (95% CI) | 0.0542 (0.0498–0.0590) | 0.0481 (0.0463–0.0499) | 0.0476 (0.0468–0.0485) | 0.0458 (0.0448–0.0467) | 0.0515 (0.0502–0.0528) |
| Mean (SD) LoS per hHF event | 5.8 (12.5) | 5.2 (5.1) | 5.2 (5.8) | 5 (5.9) | 5.4 (6.3) |
| Mean (SD) cumulative LoS | 8.4 (17.9) | 7.2 (8.6) | 6.7 (7.9) | 6.6 (8.1) | 7.2 (9.1) |
| **Mean (SD) cost, $** |  |  |  |  |  |
| All medication (all patients) | 10,935 (44,315) | 13,867 (44,205) | 9,746 (24,444) | 10,526 (32,976) | 10,992 (28,738) |
| HF medication (resource users) | 1,248 (2,994) | 1,950 (10,830) | 1,981 (3,906) | 2,054 (6,722) | 1,785 (5,131) |
| Outpatient visits (all patients) | 50,374 (153,200) | 48,943 (134,433) | 36,086 (90,094) | 36,102 (97,034) | 44,687 (116,753) |
| Urgent visits (resource users) | 2,091 (3,536) | 1,850 (3,519) | 978 (2,469) | 1,185 (2,665) | 1,088 (2,762) |
| Inpatient stays (all cause) | 70,059 (211,820) | 62,168 (146,733) | 31,321 (70,959) | 36,662 (92,102) | 45,309 (121,328) |
| hHFs (resource users) | 72,918 (278,011) | 35,844 (77,260) | 18,190 (36,921) | 2,650 (20,968) | 2,479 (28,230) |

Abbreviations: CI, confidence interval; HF, heart failure; hHF, heart failure hospitalization; LoS, length of stay; SD, standard deviation.

*P*<0.05 for all comparisons across ejection fraction subgroups with the following exceptions: number of inpatient stays according to age (*P*=0.6978); number of urgent visits according to age (*P*=0.234); mean cumulative LoS (in resource users) according to age (*P*=0.1328); cost of urgent visits (all patients) according to prior hospitalization (*P*=0.6058); and cost of hHFs (resource users) according to prior hospitalization (*P*=0.4198).

**SUPPLEMENTARY TABLE S2** Healthcare resource utilization and direct costs during the follow-up period by diagnosis of type 2 diabetes mellitus or chronic kidney disease at indexing

|  | T2DM | | CKD | |
| --- | --- | --- | --- | --- |
| Resource | No (n=64,774) | Yes (n=44,947) | No (n=85,957) | Yes (n=23,764) |
| **All-cause resource use**  **Outpatient** |  |  |  |  |
| No. of outpatient visits, mean (SD) | 62.8 (77.7) | 84.8 (107.2) | 64.2 (71.1) | 99.2 (139.7) |
| Incidence rate  (95% CI) | 2.77 (2.767–2.772) | 3.77 (3.762–3.770) | 2.764 (2.762-2.767) | 4.875 (4.869–4.882) |
| **Inpatient** |  |  |  |  |
| No. of hospital admissions, mean (SD) | 1.4 (1.8) | 1.8 (2.3) | 1.5 (1.9) | 1.9 (2.4) |
| Incidence rate  (95% CI) | 0.062 (0.062–0.063) | 0.080 (0.079–0.080) | 0.064 (0.063–0.064) | 0.094 (0.094–0.095) |
| Mean (SD) LoS/hospitalization | 5.9 (6.1) | 6.2 (6.6) | 5.9 (6.2) | 6.5 (6.4) |
| Mean (SD) cumulative LoS | 12.9 (19.9) | 16.2 (24.2) | 13.5 (20.6) | 17.3 (25.5) |
| **HF-related resource use** |  |  |  |  |
| Urgent visits (all patients) |  |  |  |  |
| Mean no. (SD) | 0.1 (0.5) | 0.2 (0.6) | 0.1 (0.5) | 0.2 (0.6) |
| Incidence rate  (95% CI) | 0.005  (0.005-0.005) | 0.007  (0.007-0.008) | 0.006  (0.005-0.006) | 0.009  (0.008-0.009) |
| Urgent visits (resource users) | *n=5,220* | *n=4,780* | *n=7,261* | *n=2,739* |
| Mean no. (SD) | 1.5 (1.1) | 1.6 (1.2) | 1.5 (1.1) | 1.5 (1.2) |
| Incidence rate  (95% CI) | 0.056  (0.055-0.058) | 0.055  (0.054-0.056) | 0.055  (0.054-0.056) | 0.058  (0.056-0.060) |
| hHFs (all patients) |  |  |  |  |
| Mean no. (SD) | 0.1 (0.4) | 0.2 (0.5) | 0.1 (0.5) | 0.2 (0.5) |
| Incidence rate  (95% CI) | 0.005  (0.005-0.006) | 0.008  (0.008-0.008) | 0.006  (0.006-0.006) | 0.009  (0.009-0.009) |
| hHFs (resource users) | *n=6,431* | *n=5,821* | *n=8,926* | *n=3,326* |
| Mean no. (SD) | 1.2 (0.6) | 1.3 (0.8) | 1.3 (0.7) | 1.3 (0.7) |
| Incidence rate  (95% CI) | 0.048  (0.047-0.049) | 0.048  (0.047-0.049) | 0.047  (0.046-0.048) | 0.051  (0.049-0.052) |
| Mean (SD) LoS (all hHF events) | 5.0 (5.2) | 5.4 (6.8) | 5.1 (6.1) | 5.5 (5.9) |
| Mean (SD) cumulative LoS | 6.4 (7.8) | 7.3 (9.3) | 6.6 (8.6) | 7.3 (8.2) |
| **Direct medical costs, mean (SD) $** |  |  |  |  |
| Medication, all | 8,762 (30,195) | 13,559 (32,517) | 10,383 (31,944) | 11,953 (28,598) |
| HF medication |  |  |  |  |
| All | 1,464 (3,432) | 1,739 (7,637) | 1,633 (6,049) | 1,375 (3,180) |
| Resource users | 1,840 (3,757) | 2,079 (8,308) | 2,019 (6,668) | 1,666 (3,430) |
| Outpatient visits, all | 34,610  (88,467) | 47,110 (126,519) | 33,062  (61,671) | 63,851  (193,091) |
| Urgent visits |  |  |  |  |
| All | 97 (854) | 115 (919) | 101 (866) | 115 (934) |
| Resource users | 1,198 (2,782) | 1,078 (2,627) | 1,195 (2,753) | 997 (2,587) |
| Inpatient stays, all | 38,301  (108,359) | 43,222  (101,253) | 38,787  (104,207) | 45,850  (110,019) |
| hHFs |  |  |  |  |
| All | 2,338 (26,729) | 2,923 (20,230) | 2,466 (22,360) | 2,981 (30,307) |
| Resource users | 23,550 (81,838) | 22,571 (52,334) | 23,749 (65,649) | 21,300 (78,575) |
| Total | 81,672  (163,754) | 103,881 (189,061) | 82,232 (145,120) | 121,654  (252,698) |

Abbreviations: CI, confidence interval; CKD, chronic kidney disease; HF, heart failure; hHF, heart failure hospitalization; LoS, length of stay; NEC, not elsewhere classified; SD, standard deviation; T2DM, type 2 diabetes mellitus.

*P*<0.05 for all comparisons across T2DM and CKD subgroups with the exception of: number of urgent HF visits in resource users (T2DM), number of hHFs in resource users (T2DM), hHF average and cumulative LoS (T2DM), urgent HF visit costs (T2DM), urgent HF visit costs in resource users (CKD), inpatient stay costs (T2DM), hHF costs (CKD), hHF costs in resource users (CKD).

**SUPPLEMENTARY FIGURE S1** Sample generation flow diagram


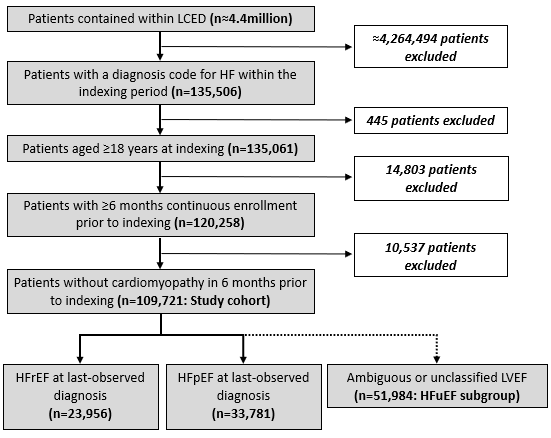


HF, heart failure; LCED, Limited Claims-Electronic Health Record Dataset; HFpEF, heart failure with preserved ejection fraction; HFrEF, heart failure with reduced ejection fraction; HFuEF, heart failure with unclassified ejection fraction

**SUPPLEMENTARY FIGURE S2** Medication use in follow-up period: (A) GDMT; (B) other concomitant medications. *P*<0.05 for all HFrEF/HFpEF comparisons with the following exception: SGLT-2s

A


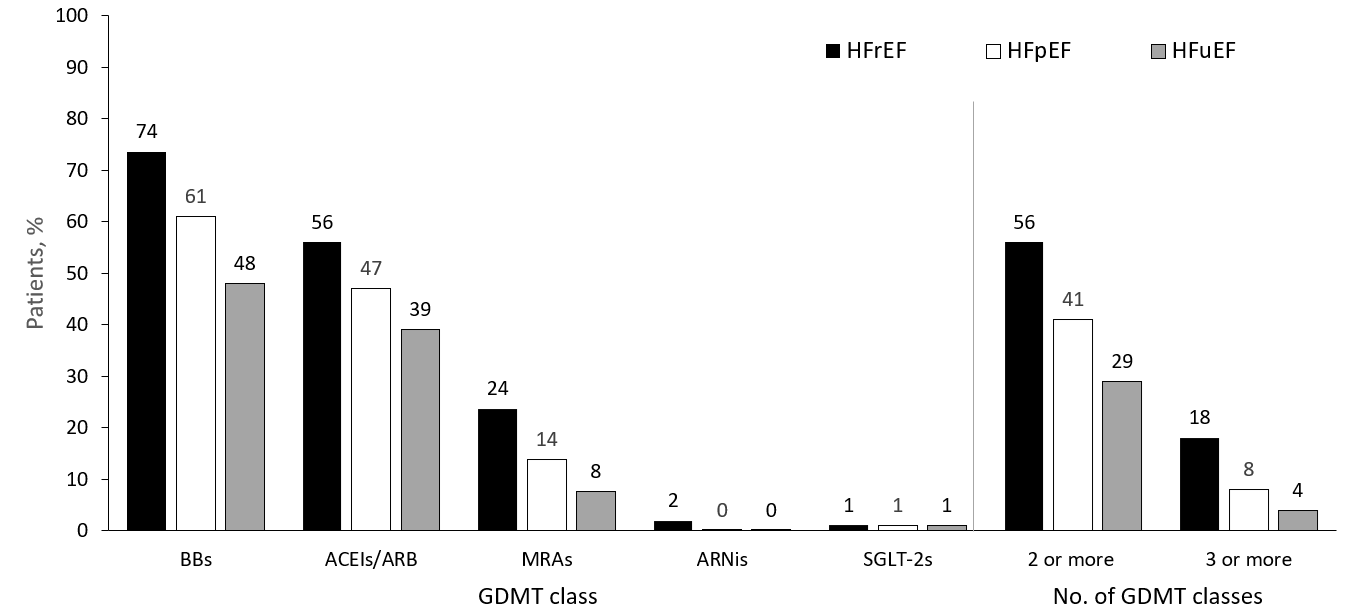


B


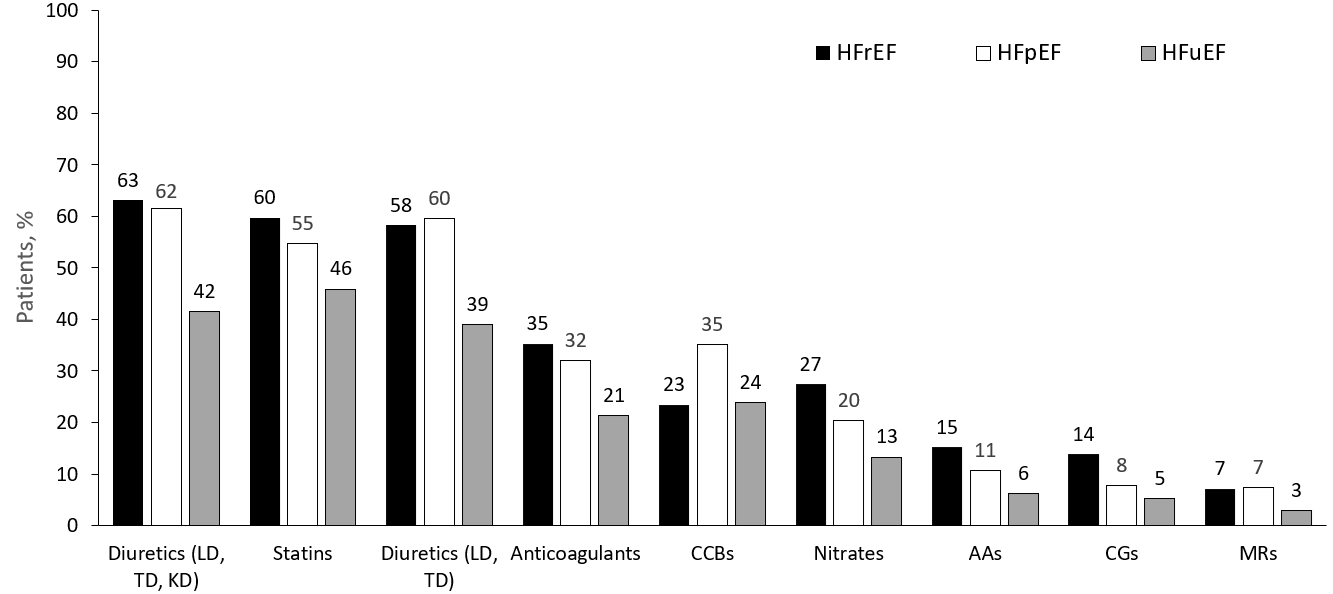


AA, anti-arrhythmic; ACEI, angiotensin-converting enzyme inhibitor; ARB, angiotensin receptor blocker; ARNI, angiotensin receptor-neprilysin inhibitor; BB, beta-blocker; CCB, calcium channel blocker; CG, calcium glycoside; GDMT, guideline-directed medical therapy; HFpEF, heart failure with preserved ejection fraction; HFrEF, heart failure with reduced ejection fraction; HFuEF, heart failure with unclassified ejection fraction; KD, potassium diuretic; LD, loop diuretic; MR, muscle relaxant; MRA, mineralocorticoid receptor antagonist; SGLT-2, sodium-glucose cotransporter-2 inhibitor; TD, thiazide diuretic

**SUPPLEMENTARY FIGURE S3** All-cause costs across the entire follow-up period


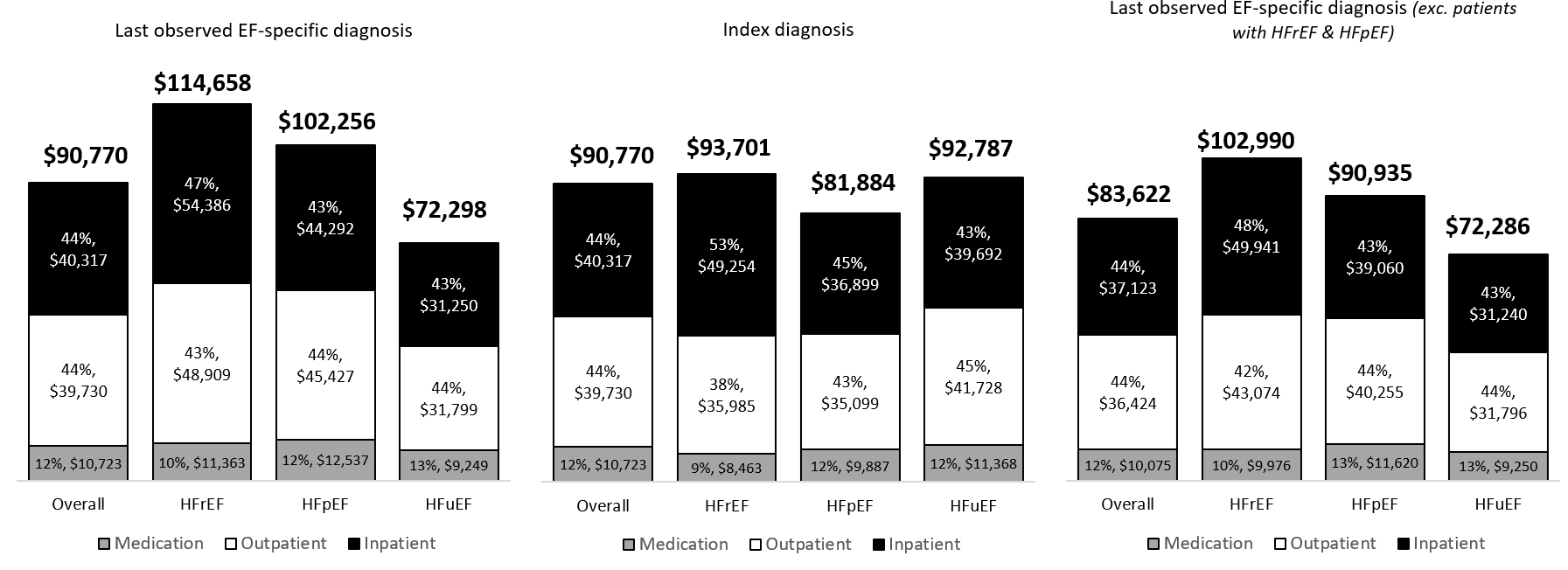


EF, ejection fraction; HFpEF, heart failure with preserved ejection fraction; HFrEF, heart failure with reduced ejection fraction; HFuEF, heart failure with unclassified ejection fraction
